# Automatic classification of histopathological diagnoses for building a large scale tissue catalogue

**DOI:** 10.1007/s12553-016-0169-8

**Published:** 2016-12-22

**Authors:** Robert Reihs, Heimo Müller, Stefan Sauer, Kurt Zatloukal

**Affiliations:** Medical University of Graz, A-8010 Graz, Austria

**Keywords:** Automatic classification, Text mining, Decision Trees, Biobank

## Abstract

In this paper an automatic classification system for pathological findings is presented. The starting point in our undertaking was a pathologic tissue collection with about 1.4 million tissue samples described by free text records over 23 years. Exploring knowledge out of this “big data” pool is a challenging task, especially when dealing with unstructured data spanning over many years. The classification is based on an ontology-based term extraction and decision tree build with a manually curated classification system. The information extracting system is based on regular expressions and a text substitution system. We describe the generation of the decision trees by medical experts using a visual editor. Also the evaluation of the classification process with a reference data set is described. We achieved an F-Score of 89,7% for ICD-10 and an F-Score of 94,7% for ICD-O classification. For the information extraction of the tumor staging and receptors we achieved am F-Score ranging from 81,8 to 96,8%.

## Introduction

Many hospitals and medical universities have a large medical data pool, that they have acquired throughout the last years. Knowledge embedded in such data collections contains information of great relevance for biomedical research. The information can be utilized for biobanking, epidemiology or public health planning and evaluation. Exploring knowledge in these “big data” pools is a very challenging task, especially when dealing with inhomogeneous data collections built over several decades with evolving information. To utilize the assets, it is necessary to search and analyze the knowledge in a structured homogeneous way.

In smaller projects with less than 100 patients it is possible to do the data cleanup manually. However, with hundreds of thousands of patients, as needed for epidemiological studies, when converting clinical collections to a biobank or to analyze the public health system, an automated preprocessing system is needed.

The starting point of our undertaking is the tissue collection of the Institute of Pathology. The Dataset contains approximately 1.4 million samples from 700.000 patients recorded over 23 years with a minimum of 5 years of follow-up data. This cohort represents a non-selected patient group characteristic for Central Europe, which is now part of the Biobank of the Medical University of Graz [1] and part of the Central Research Infrastructure for Molecular Pathology (CRIP) [2]. The scientific value of the tissue collection is not only characterized by the size and its technical homogeneity of processing, but also by its population-based character. These features provide amongst others ideal opportunities for epidemiological studies, and health care system evaluation, and allow the validation of biomarkers for identification of specific diseases and the response to treatment regimes. These collations can also have an impact on the transformation to personalized medicine.

The alteration of terms in past years, different ontologies, synonyms, the change in classification, misspellings in the text and different descriptions of clinical findings pose the main challenges in this area. These problems occur especially with data covering a long time period and a highly dynamic field like medicine. To extract well-structured medical information from unstructured plain text is a difficult task. We therefore created a set of tools SAAT (Semi-Automated Annotation Tool), to classify the findings and extract information from the unstructured texts.

## Methods

Our text mining tolls are a bundle of modules for cleaning up, structuring and coding clinical data records. We use a dictionary-based, decision tree text mining approach with special negation rules. An information extraction system, based on regular expressions, is used to extract the tumor staging and the receptors from the findings.

The first step in the system is to correct misspellings and abbreviations to get a clean diagnosis field. This step is only necessary at import time of the data. The next step is to merge different diagnoses together, because in some clinical cases there are several findings extracted from the same case. Only the merged case has all the information relevant for the diagnosis of the patient. The last step is the coding and extraction of the information from the diagnosis. We classify medical records according to the ICD-10 (International Classification of Diseases 10th revision) [3] and ICD-O-3 (International Classification of Diseases for Oncology 3rd revision) [4] classification systems and extract information about the tumor staging and the receptors. The result of the classification is stored in the relational database or sent as a result in the case of a service call.

### Data cleanup

The integration of data sources into the relational database of the system is the initial step for using the complete tool set. If the text mining system is used as a service this step is not necessary, but then not all tools are available. In the integration step existing data are stored in the database schema with a link to the original source. Patients can be merged together with a separate tool, not necessary in the text mining step, called patient merger. The individual findings are merged together to diagnosis. This can be necessary when part of the diagnosis is coming in a later stage of the diagnosis step. For specific fields we generate dictionaries. For example, all physician names even if misspelled or abbreviated, are mapped to a physician dictionary. In the case of physicians, a system for coding of the title was developed, because these usually change over time.

### Information extraction

In the information extraction module, we use a regular expression approach with a substitution system, to extract information about the T (Tumor dimension), N (Lymph node), M (Metastases), G (Grading), R (Resection boundaries), L (Invasion into lymphatic vessels), and V (Invasion into vein) staging and organ receptors such as progesterone receptor, estrogen receptor, fish, HER2/neu. The extraction systems are regular expression based, but can also handle textual descriptions of the staging or the receptor. For textual description in staging, there could be for example the text “DIE RESEKTION ERFOLGTE DER TIEFE ZU NICHT IM GESUNDEN” (The basal resection margin was tumor positive) which is translated to R 1. For receptors there is the additional problem of a textual grouping and an exact numeric value. All combinations of usage are possible. As an example for mamma carcinoma the estrogen and progesterone receptor status can be specified with: 0 means negative; <10%, 1, 2 and 3 means mildly depressed; 4 and 6 means moderately depressed; 8, 9 and 12 means severely depressed. In the case of only a textual description, a value is assigned for the group, and the record is marked in the database as “automated assigned”, to filter for the values if the exact value is needed for a search question.

### Classification

The core module of the system is the classification module [5]. In the text preparation step certain words are merged to terms to underline their concept for the classification. For example, such a term in our representation of pathological data is “Metastase eines” (metastasis of a) which in this case means that the following tumor described is not a primary carcinoma, but a metastasis of another tumor. In this preparation step, the text is also split into single terms (tokenized), left and right neighbors, sentences and findings.

In our text mining approach, we use a decision tree based system. Every node of the classification tree represents a matching word, described by a regular expression pattern, for different spellings and synonyms (tumor and carcinoma). Additionally, every node has a set of processing rules. These rules contain flags about the valid position of the terms: foreword, ending, in sentence, in finding, in the whole case, Xth left neighbor, Yth right neighbors. In the rules also negations, code type and value and priorities are defined. The module of the classification tree can be separated into a different storage location. The dictionary can then be stored in a central database system. With this setup a corporate classification tree between multiple institutions, working in the same language, can be setup to minimize the effort for maintaining and updating the classification rules for each group. Only the classification tree is shared and no medical data are exchanged, so there is no privacy issue with this setup. To consider lingual differences between the institutions, institutional rules can be specified for different locations. In case of a separate location of the dictionary a cashing database is implemented, to be able to run the classification also when the centralized location is not reachable.

**Fig. 1:**
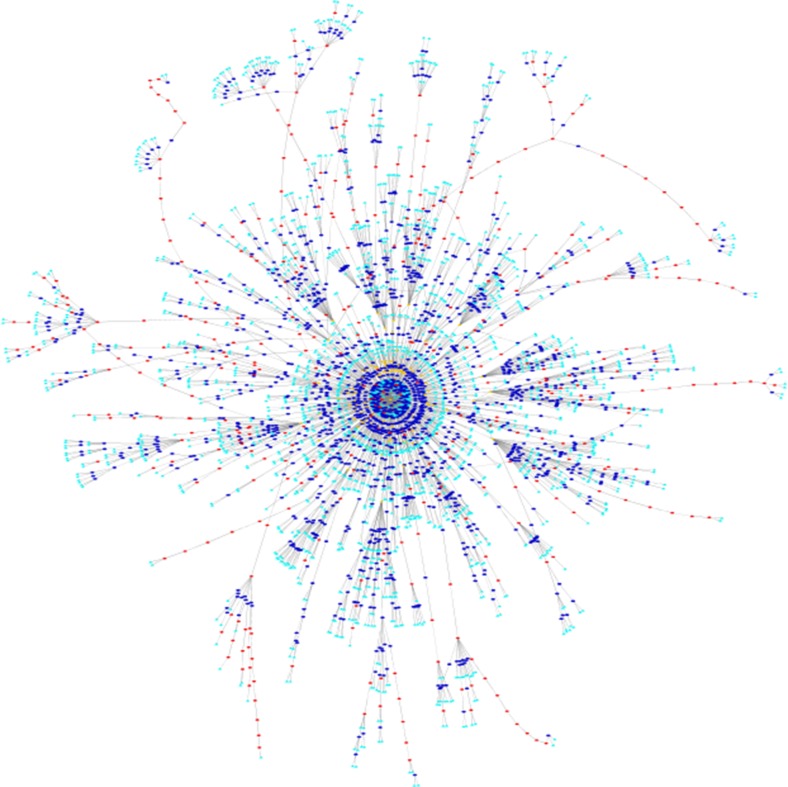
104 classification trees with an overall number of 4285 nodes Created with Cytoscape

**Fig. 2:**
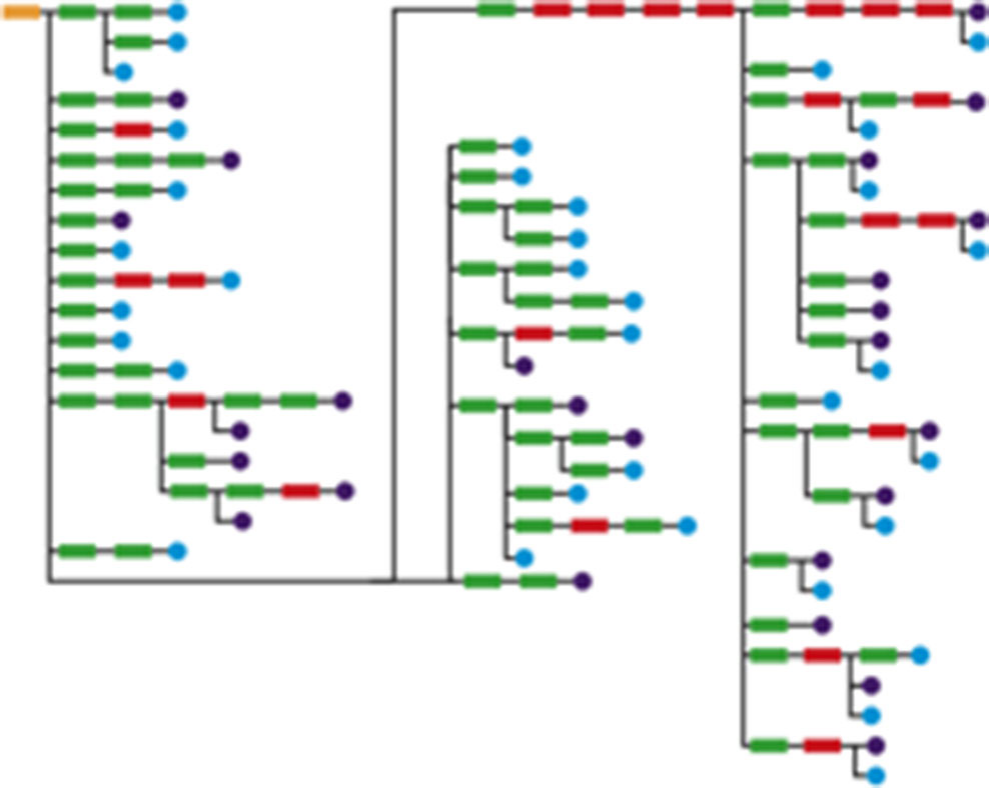
Classification tree for mamma carcinoma Created with Photoshop

Currently we have a set of 104 classification trees with an overall number of 4285 nodes shown in Fig. 1. These classification trees were created with the help of an interactive tree editor and an ontology-based term extraction tool by medical experts at the Institute of Pathology in Graz. Figure 2 shows the outline of the complete tree for the classification of ICD-10 codes and ICD-O codes related to mamma carcinoma. The decision tree consists of the start (root) node, rule nodes and negation nodes. The resulting classification can be either ICD-10 code nodes or ICD-O code nodes.

**Fig. 3:**
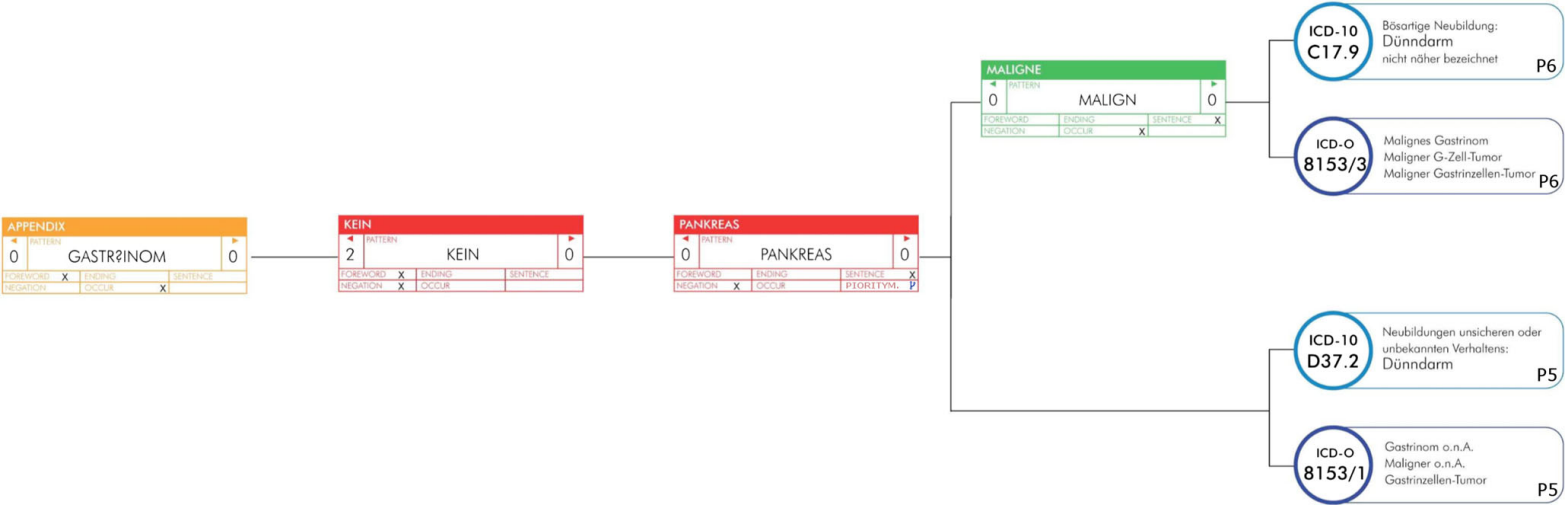
A single branch of a decision tree with nodes showing the rules for the tree with the entry point of the synonym of “Gastrinoma”. Created with Photoshop

In Fig. 3 a single branch of a decision tree is shown. The node shows the rules for the tree with the entry point of the synonym of “Gastrinoma”. The color of the node depicts the node type: root node (orange), rule node (green), negation node (red). The pattern is a regular expression to match different spellings of the synonym. The number on the left and right side of the pattern indicate the left and right neighbors this node can occur in relation with the previse node that was found in the text. The negation node with the synonym “Pankreas” acts as a priority merging node for the coding. All codes matching this part of the classification tree are merged on this point with their priorities. In this case, the codes following the synonym “Malign” have a priority of 6 and the other branch codes have a priority of 5 indicated in the right bottom corner of the code. That means, if the codes (propriety 6) ICD-10: C17.9 (Malignant neoplasm of small intestine, unspecified) and ICD-O: 8153/3 (Gastrinoma, malignant) have a hit, the codes (priority 5) ICD-10: D37.2 (Neoplasm of uncertain behavior of small intestine) and ICD-O: 8153/1 (Gastrinoma, NOS) are discarded.

## Results

### Classification

The result of the classification system is evaluated by medical experts. Additionally, medical experts created a manually coded reference data set. For the evaluation we created a web based tool for the experts that can also track the changes between classification runs. In addition, the reference data set is calculated after every classification run and is visualized in a web tool. In this tool the “hit rate” is visualized. For each run of the classification system the results are stored for a later comparison. In the classification we achieved for the ICD-10 codes an F-Score of 89,7% (precision 83,2% and recall 97,5%) and for the ICD-O codes an F-Score of 94,7% (precision 91,2% and recall 98,5%). Table 1 shows the F-Score values for the extraction of the tumor staging. In comparison we compute the F-Score for a simple classification at the lexical level with regular-expressions using the “fruit machine” method [6]. The column “FM original” shows the results using the original records and the column “FM corrected” the F-Scores of the regular-expression classification on the preprocessed data. Our extraction method shows a very good performance, compared to others. It could be further improved by extending the cleanup for textual description of the tumor stated or additional graph-based extraction methods [7].

**Table 1:** Precision, recall and F-Score values for the classification of tumor staging

			FM original		FM corrected		Classification	
T Staging	Precision	F-Score	74,4%	53,6%	55,6%	48,6%	96,2%	95,0%
	Recall		41,9%		43,1%		93,8%	
N Staging	Precision	F-Score	70,6%	38,5%	40,6%	40,1%	84,3%	85,5%
	Recall		26,5%		39,7%		86,8%	
M Staging	Precision	F-Score	60,0%	20,5%	47,2%	49,8%	81,2%	82,8%
	Recall		12,4%		52,6%		84,5%	
G Grading	Precision	F-Score	81,6%	48,6%	77,6%	49,1%	94,6%	96,8%
	Recall		34,6%		35,9%		99,1%	
R Staging	Precision	F-Score	61,1%	17,2%	21,0%	22,2%	77,0%	84,9%
	Recall		10,0%		23,6%		94,5%	
L Staging	Precision	F-Score	62,5%	33,3%	14,3%	18,8%	81,8%	81,8%
	Recall		22,7%		27,3%		81,8%	
V Staging	Precision	F-Score	100%	12,5%	100%	12,5%	100%	92,9%
	Recall		6,7%		6,7%		86,6%	

### Example data record

Input for the data cleanup is shown in Table 2, describing 2 diagnoses for one patient, where in one of the entries the name of the patient is misspelled.

**Table 2:** Input for data cleanup

Patient name	Date of birth	Date of diagnosis	diagnosis
Graller Violetta	03.02.1985	14.05.1999	LOW DIFFERENTIATED INVASIVE DUCTAL MAMMA CACINOMA (NOS, 3,5 CM MAX: DIAMETER, MINIMAL RESECTION DISTANCE AFTER BASAL 5 MM). PT 2 15 METASTASES FREE LYMPHNODES. NON-INVASIVE CARCINOMA COLLECTIONS IN THE SINUS LACTIFERI. RESECTIONBOUNDARIES FREE OF CANCER. GRADING 3, N 0.
Graller Violeta	03.02.1985	17.05.1999	ESTROGENRECEPTOR: STRONG (SCORE 12) PROGESTERONRECEPTOR: STRONG

In the Data Cleanup step the diagnosis is merged to one diagnosis, the misspelling of the patient name is corrected. Also the misspellings in the text are corrected, in this case the CACINOMA is set to CARCINOMA.

In the preprocessing of the Information Extraction module fist the textual descriptions are mapped to a standardized format. In the example RESECTIONBOUNDARIES FREE OF CANCER is mapped to R-0 and GRADING 3 to G-3. The extraction of the stagings is then handled by the regular expression module. The result is shown in Table 3. For the receptors the values are extracted and the missing value of the progesterone receptor is set to a numeric value and marked as automated assigned.

**Table 3:** Result data stored in Data warehouse

Result Data Cleanup			
Patient name	Date of birth	Date of diagnosis	Diagnosis
Graller Violetta	03.02.1985	14.05.1999	LOW DIFFERENTIATED INVASIVE DUCTAL MAMMA CARCINOMA (NOS, 3,5 CM MAX: DIAMETER, MINIMAL RESECTION DISTANCE AFTER BASAL 5 MM). PT 2 15 METASTASES FREE LYMPHNODES. NON-INVASIVE CARCINOMA COLLECTIONS IN THE SINUS LACTIFERI. RESECTIONBOUNDARIES FREE OF CANCER. GRADING 3, N 0; −----ESTROGENRECEPTOR: STRONG (SCORE 12) PROGESTERONRECEPTOR: STRONG
Result Information Extraction			
Diagnosis (preprocessing)	(MAMMA) LOW DIFFERENTIATED INVASIVE DUCTAL MAMMA CARCINOMA (NOS, 3,5 CM MAX: DIAMETER, MINIMAL RESECTION DISTANCE AFTER BASAL 5 MM). PT 2		
	15 METASTASES FREE LYMPHNODES. NON-INVASIVE CARCINOMA COLLECTIONS IN THE SINUS LACTIFERI. RESECTIONBOUNDARIES FREE OF CANCER R-0. GRADING 3, G-3, N 0; ESTROGENRECEPTOR: STRONG (SCORE 12) PROGESTERONRECEPTOR: STRONG		
Data stored	T = 2	G = 3	*N* = 0
	R = 0	ESTROGENRECEPTOR = (STRONG, 12)	PROGESTERONRECEPTOR = (STRONG, 9*(auto assigned))
Result Classification			
ICD-10 = C50.9	Malignant neoplasm of breast of unspecified site	ICD-O = 8500/3	Infiltrating duct carcinoma, NOS

The ICD-10 and ICD-O classification is extracted the preprocessed diagnosis. The used subtree for the classification states with “MAMMA” as the root node. The classification system hit on “CARCINOMA”, “INVASIVE” and “DUCTAL”. Additionally, there where 6 negation nodes not hit. The result of the route in the subtree are two leaf nodes for ICD-10 C50.9 and ICD-O 8500/3.

### Literature comparison

We compared the tumor distribution of our classification tool with the distribution described in the respective text books for pathology [8–10] and international statistic of the NCI [11]. The results of the comparison are shown in Table 4.

**Table 4:** Comparisons of mamma carcinoma distribution in text books for pathology and statistic reports

	Text mining Tool	W. Remmele, Pathologie [8]	Harris JR, Diseases of the Breast [9]	Böcker/Denk/Heitz, Pathologie [10]	NCI, Cancer Statistics Review [11]
Ductal Ca	78,9%	67,9%	65–80%	ca. 80%	67,6%
Lobular Ca	11,2%	6,3%	5–10%	10–20%	8,0%
Medullary Ca	2,0%	2,8%	<5%	<1%	0,7%
Mucinous Ca	3,6%	2,2%	<2%	2%	2,5%
Tubular Ca	2,4%	0,7%	1%	1–2%	1,6%
Papillary Ca	1,9%	0,9%	<2%	<1%	0,6%

### Time calculations manual vs automated

For a showcase, we evaluated the result of the text mining classification of colon findings within 1 year. In the test year we have 4215 colon findings. After an initial training phase, to get familiar with the colon diagnosis and the ICD-10 and ICD-O codes for this organ, we stopped the time for evaluating 50 findings. The 50 findings contained 35 non cancer findings and 15 cancer findings. We did 5 cycles of 50 findings and obtained an average of 53 min for coding 50 findings. Than we calculated the time needed for manually coding the year 2000, this would take 77 h. To manually code the dataset from the pathology in Graz from 1984 to 2011, it would take around 2253 h for the 122896 findings.

### Runtime testing

The classification process is performed with a linear processing time. The loading of the dictionary always needs constant time. This is especially important when running the system as a service, because this can increase the response time significantly when the dictionary is not loaded on request. In the schema only modified fields are updated to minimize the database interactions. The software is implemented using java with the possibility to run the classification in a multithreaded environment, to scale for big datasets. The run time analysis was performed on a dual core system with 1.67 GHz with 2GB RAM running Windows XP, shown in Table 5. The classification part is divided into a static part (Initializing dictionary) and a dynamic part (Text Mining, DB updates).

**Table 5:** Module run time, performed on a dual core system with 1,67GHz, 2GB RAM on Windows XP

Number of Findings	Pool together	Spell correction	DB update	Initializing dictionary	Text Mining	DB update
1. run/1 k	0,7–0,9 sec	0,4–0,6 sec	0,6–1,3 sec	20–30 sec	0,3–0,6 sec	0,5–1,4 sec
2.runs/1 k	0,7–0,9 sec	0,4–0,6 sec	0–1,3 sec	20–30 sec	0,3–0,6 sec	0–1,4 sec
1. run/10 k	7–8,5 sec	4–5 sec	5,2–12,6 sec	20–30 sec	3,5–7,3 sec	5–12,8 sec
2. runs/10 k	7–8,5 sec	4–5 sec	0–12,6 sec	20–30 sec	3,5–7,3 sec	0–12,8 sec

### Showcases

**Fig. 4:**
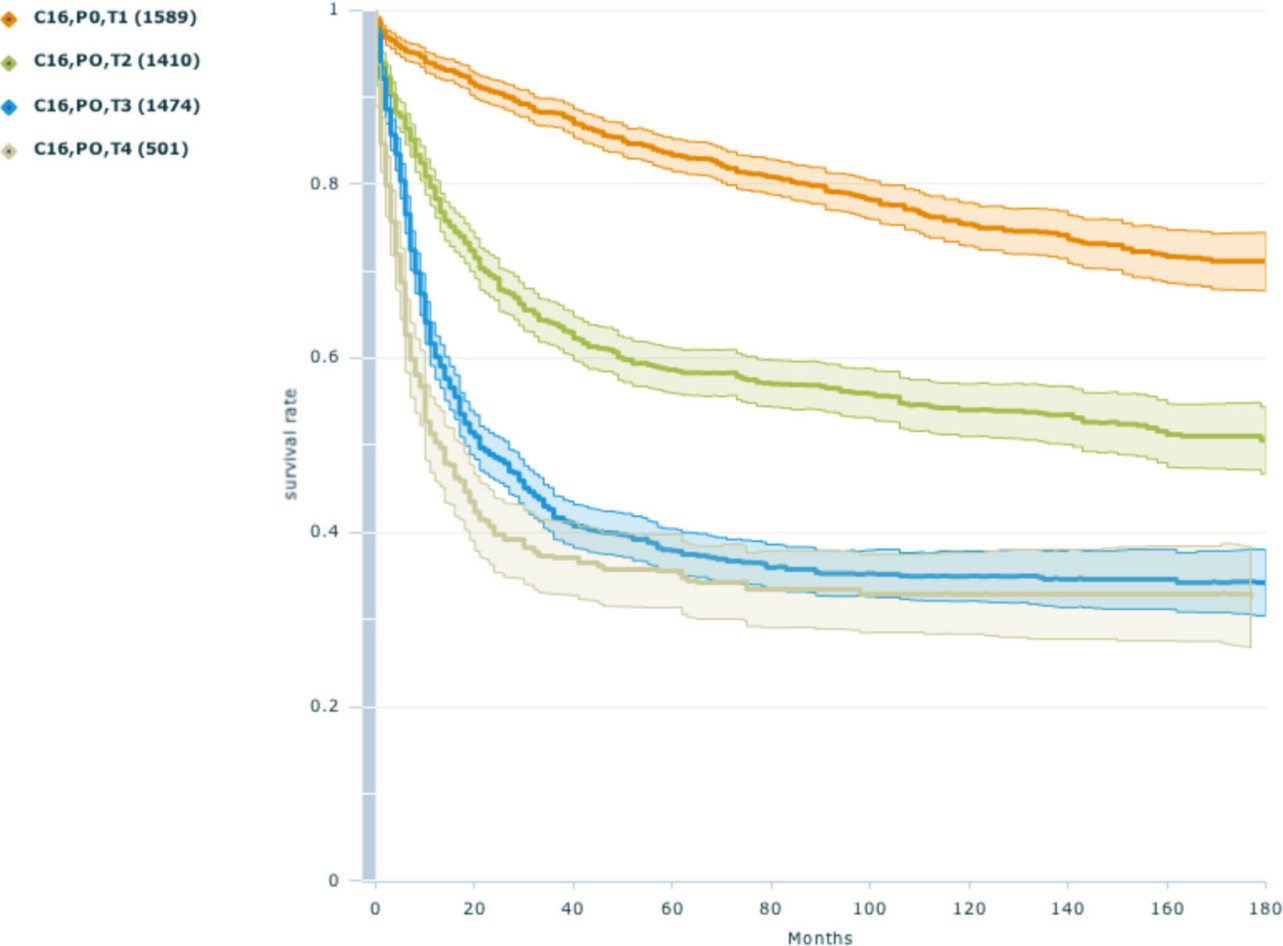
Overall survival of patients with an operation of a Malignant neoplasm of stomach with different tumor staging’s. Created with WebTool MedicalExplorer

**Fig. 5:**
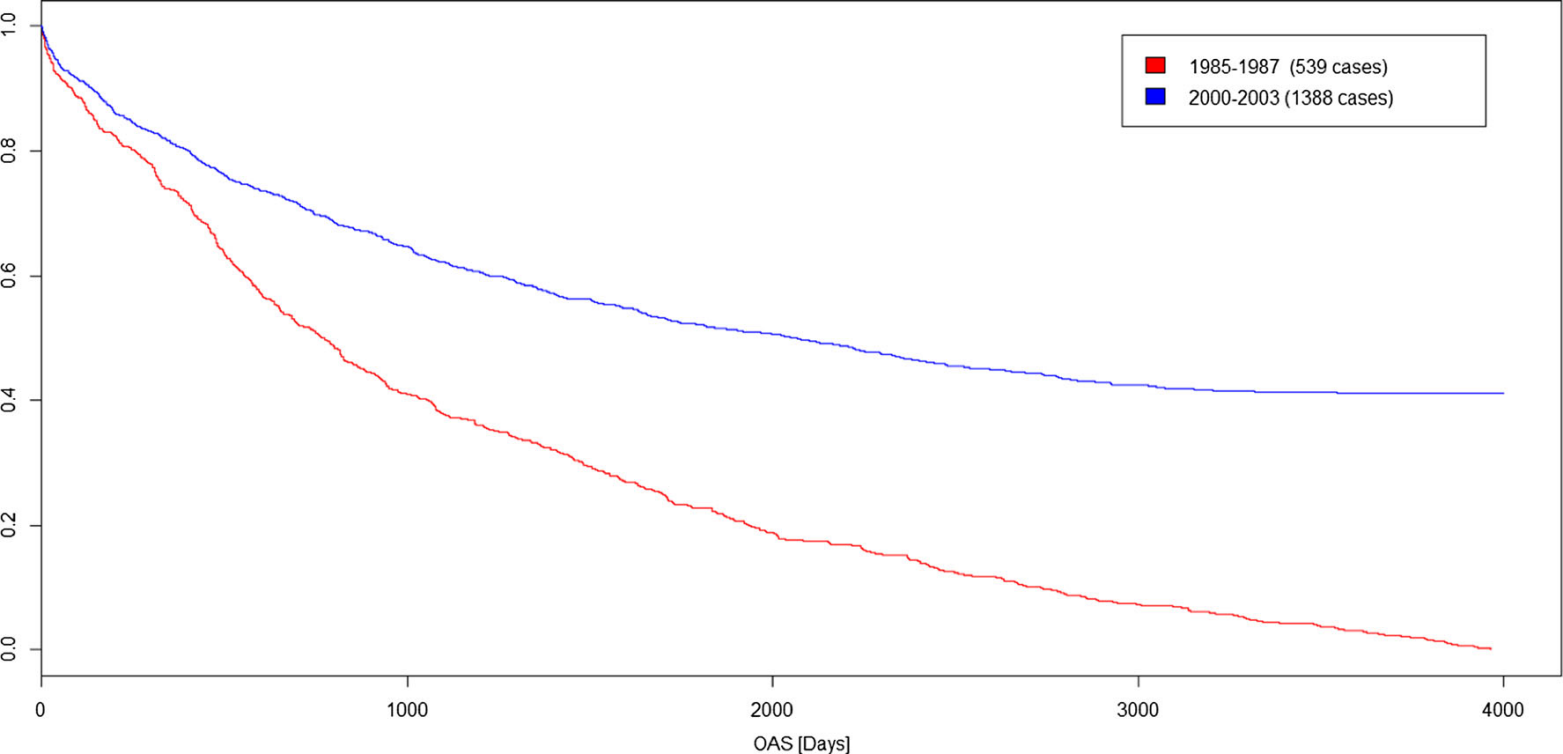
Survival of Colon cancer patients diagnosed between 1985 to 1987 and 2000 and 2003 Created with R

In this section we showcase some analysis done with the data classified by our tool. Figure 4 shows the difference in overall survival of patients with an operation of a malignant neoplasm of the stomach. The graph shows the difference in survival with different tumor staging T for 4974 patients. As expected, patients with higher T staging have a shorter overall survival compared to patients with a low T staging. To showcase an improvement of the healthcare system, in Fig. 5 the survival of colon cancer patients with a difference of 15 years between the date of operation is compared. It shows clearly that the patients operated between 1985 to 1987 have a significantly lower survival expectation than patients operated between 2000 and 2003. The classification and extraction result can be used to create complex statistics and graphs for a large number of cases [12].

## Discussion

We developed an NLP software for pathology based on ontology-based term extraction and a semi-manually curated decision tree. With our approach we achieved in the coding of real world data sets for the ICD-10 codes an F-Score of 89,7% (precision 83,2% and recall 97,5%) and for the ICD-O codes an F-Score of 94,7% (precision 91,2% and recall 98,5%). For the extraction of tumor staging we achieved an F-Score of 81,8%–96,8%.

Comparing the result with other state of the art approaches shows a little higher accuracy. This is due to the fact that our system is specially tailored to German pathology diagnosis and has a manually optimized classification tree. Fully automatic NLP methods can be easily trained to new tasks, when trainings sets are available [13]. A study on classification of free-text death certificates shows an F-Score of 94,2% [14] for classification of cancer and an F-Score of 70% for the ICD-10 classification. In the medical NLP challenge the best score for the classification achieved a F-Score of 89% [15]. The classification tree currently models the semantics of German pathological findings, however the rule sets can easily be translated into other languages and adapted to additional coding systems, e.g. SNOMED. In addition automated methods for generating a translated version of the classification tree can be used and evaluated.

Piwowar [16] pointed out, that “Academic health centers (AHCs) have a critical role in enabling, encouraging, and rewarding data sharing. The leaders of medical schools and academic-affiliated hospitals can play a unique role in supporting this transformation of the research enterprise”. Our toolset will help medical research and public health initiatives to setup an infrastructure for biomedical data sharing. Such an undertaking is currently driven by the European research infrastructure BBMRI-ERIC [17]. In this context patient privacy is also a major aspect to be considered. Here we use the k-anonymity approach [18], which needs as prerequisite a well-structured and normalized data pool.

An additional factor in our modern society is the need in healthcare services to minimize the time a doctor must spend with a patient. On the other hand, it is impractical to provide hundreds of pages of textual information in an unstructured, unusable way to the doctor, and expect him to process all the information and register every detail. If the information is presented in a structured way, visualization techniques can help doctors obtain an overview of all medical information available in a patient’s case [19].
